# Supplementary material for: Differences in Responses of Immunosuppressed Kidney Transplant Patients to Moderna mRNA-1273 versus Pfizer-BioNTech
Source: Vaccines (Basel). 2024 Jan 17;12(1):91. doi: 10.3390/vaccines12010091 (PMC10819652; doi:10.3390/vaccines12010091)
Supplement: Supplementary file 1 [file vaccines-12-00091-s001.zip › vaccines-2693608-supplementary.pdf]

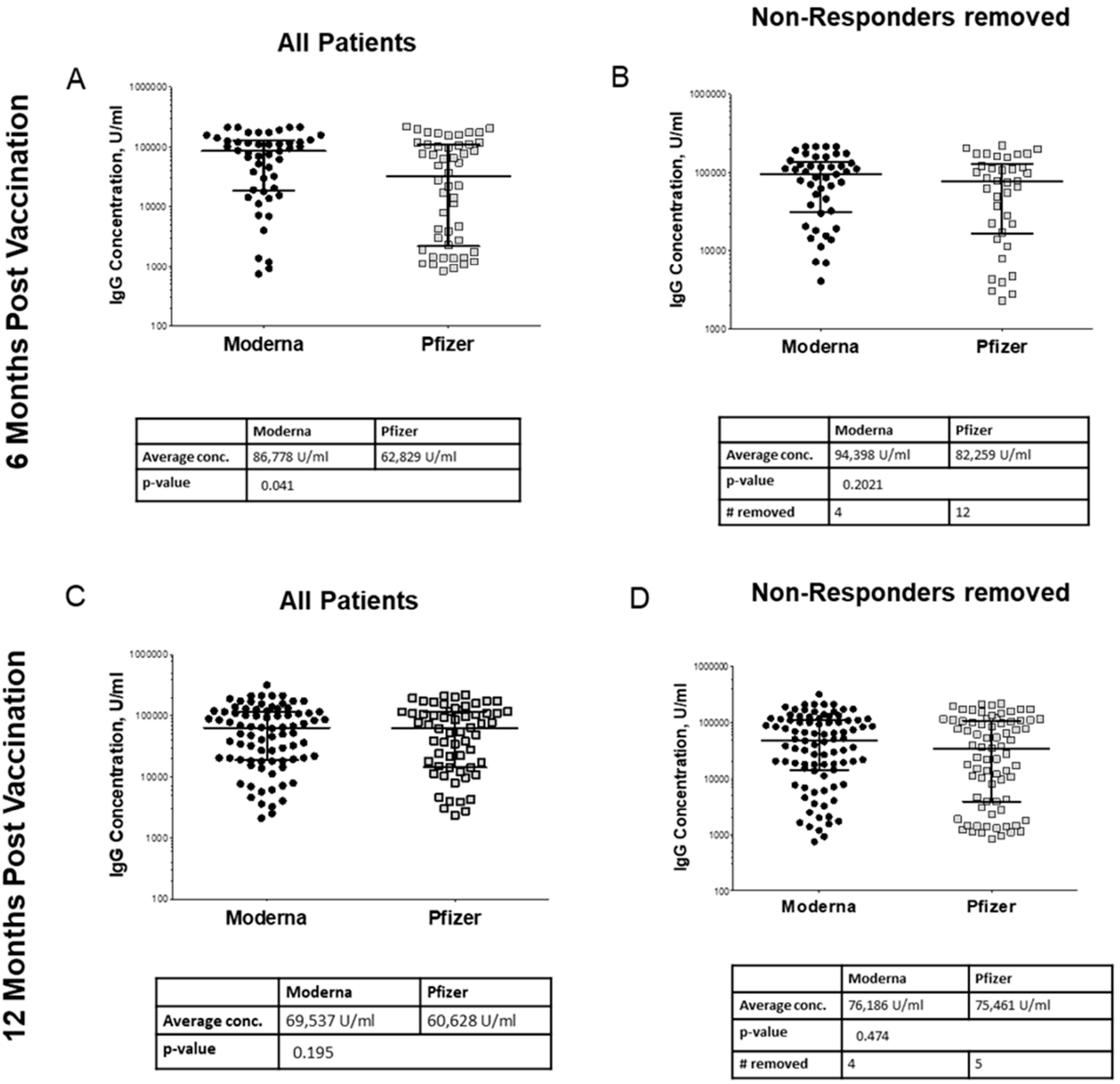

(A) 6 Months Post Vaccination – All Patients

	Moderna	Pfizer
Average conc.	86,778 U/ml	62,829 U/ml
p-value	0.041	

(B) 6 Months Post Vaccination – Non-Responders removed

	Moderna	Pfizer
Average conc.	94,398 U/ml	82,259 U/ml
p-value	0.2021	
# removed	4	12

(C) 12 Months Post Vaccination – All Patients

	Moderna	Pfizer
Average conc.	69,537 U/ml	60,628 U/ml
p-value	0.195	

(D) 12 Months Post Vaccination – Non-Responders removed

	Moderna	Pfizer
Average conc.	76,186 U/ml	75,461 U/ml
p-value	0.474	
# removed	4	5

Figure S1. Serum IgG levels in transplant recipients vaccinated with the Pfizer or Moderna vaccines in the 6-months (A and B) and 12-months (C and D) cohorts.

Summary of Results of ELISA test for IgA against SARS-CoV-2 N-protein.

Total tested	Positive	Negative	Unequivocal
144	6	136	2

Table S1. Description of the 12 months cohort.

Variable	Moderna	Pfizer	p-value*
**All Participants (n=165)**			
Race			
Caucasian	68 (75.6%)	43 (57.3%)	0.013
Other	22 (24.4%)	32 (42.7%)	
Race			
African American	12 (13.3%)	23 (30.7%)	0.007
Other	78 (86.7%)	52 (69.3%)	
Gender			
Male	45 (50.0%)	47 (62.7%)	0.103
Female	45 (50.0%)	28 (37.3%)	
Age at consent			
50 or less	34 (53.9%)	29 (46.1%)	0.439
51 or more	53 (54.6%)	44 (45.4%)	
Unknown	3 (60.0%)	2 (40.0%)	
Age at consent, years (average)	60.9	60.3	0.875
Vaccine doses			
2	59 (65.6%)	46 (61.3%)	0.575
+ boosting dose	31 (34.4%)	29 (38.7%)	
**Healthy volunteers (n=66)**			
Race			
Caucasian	29 (74.5%)	14 (51.9%)	0.059
Other	10 (25.5%)	13 (48.1%)	
Race			
African American	2 (5.1%)	6 (22.2%)	0.036
Other	37 (94.9%)	21 (77.8%)	
Gender			
Male	13 (33.3%)	13 (48.1%)	0.226
Female	26 (66.7%)	14 (51.9%)	
Age at consent			
35 or less	19 (48.7%)	17 (63.0%)	0.253
36 or more	20 (51.3%)	10 (37.0%)	
Age at consent, years (average)	63.8	64.2	0.968
Vaccine doses			
2	31 (79.5%)	17 (63.0%)	0.138
+ boosting dose	8 (20.5%)	10 (37.0%)	
**Transplant recipients (n=99)**			
Race			
Caucasian	39 (57.4%)	29 (42.6%)	0.085
Other	12 (38.7%)	19 (61.3%)	
Race			
African American	10 (45.5%)	12 (54.5%)	0.519
Other	41 (53.2%)	36 (46.8%)	
Donor type			
Living	8 (61.5%)	5 (38.5%)	0.438
Deceased	43 (50.0%)	43 (50.0%)	
Gender			
Male	32 (48.5%)	34 (51.5%)	0.394

Female	19 (57.6%)	14 (42.4%)	
BMI			
29 or less	24 (51.1%)	23 (48.9%)	
30 or more	23 (50.0%)	23 (50.0%)	0.918
Unknown	4 (66.7%)	2 (33.3%)	
Age at consent			
50 or less	15 (55.6%)	12 (44.4%)	0.622
50 or more	36 (50.0%)	36 (50.0%)	
Vaccine doses			
2	28 (49.1%)	29 (50.9%)	0.579
+ boosting dose	23 (54.8%)	19 (45.2%)	
Antimetabolites use			
Yes	39 (50.0%)	39 (50.0%)	
No	8 (50.0%)	8 (50.0%)	0.62
Unknown	4 (80.0%)	1 (20.0%)	
CNI use			
Yes	46 (49.5%)	47 (50.5%)	
No	3 (100.0%)	0 (0.0%)	0.075
Unknown	2 (66.7%)	1 (33.3%)	
Prednisone use			
Yes	39 (50.6%)	38 (49.4%)	
No	9 (50.0%)	9 (50.0%)	0.438
Unknown	3 (75.0%)	1 (25.0%)	

Table S2. Seropositivity for SARS-CoV-2 spike trimer-specific IgG in various groups of study participants in the 6 months cohort.

Group	Positive	Negative*	p-value**
**Participants at 6 months post vaccination**			
All Participants			
Healthy Volunteers	20 (74.1%)	7 (25.9%)	0.015
Transplant Recipients	33 (45.8%)	39 (54.2%)	
All Participants			
Moderna	30 (61.2%)	19 (38.8%)	0.096
Pfizer-BioNTech	23 (46.0%)	27 (54.0%)	
Transplant Recipients			
Moderna	19 (51.4%)	18 (48.6%)	0.231
Pfizer-BioNTech	14 (40.0%)	21 (60.0%)	
Healthy Volunteers			
Moderna	11 (91.7%)	1 (8.3%)	0.062
Pfizer-BioNTech	9 (60.0%)	6 (40.0%)	
**Participants at 12 months post vaccination**			
All Participants			
Healthy Volunteers	34 (51.5%)	32 (48.5%)	0.041
Transplant Recipients	40 (40.4%)	59 (59.6%)	
All Participants			
Moderna	42 (46.7%)	48 (53.3%)	0.493
Pfizer-BioNTech	32 (42.7%)	43 (57.3%)	
Transplant Recipients			
Moderna	21 (41.2%)	30 (58.8%)	0.809
Pfizer-BioNTech	19 (39.5%)	29 (60.5%)	
Healthy Volunteers			
Moderna	21 (53.8%)	18 (46.2%)	0.5628
Pfizer-BioNTech	13 (48.1%)	14 (51.9%)	

* "Negative" also includes "Indeterminate". ** Chi-squared test.
